# Application and effect of tension-reducing suture in surgical treatment of hypertrophic scar

**DOI:** 10.1186/s12893-024-02390-7

**Published:** 2024-04-23

**Authors:** Jingjing Chen, Yan Mo, Yadan Chen, Zhouji Ma, Siyun Shen, Hong Sang, Qian Tan, Ran Mo

**Affiliations:** 1 Department of Burns & Plastic Surgery, Nanjing Drum Tower Hospital, Affiliated Hospital of Medical School, Nanjing University, NO. 321, Zhongshan Road, Nanjing, China; 2 Department of Dermatology, Nanjing Drum Tower Hospital, Affiliated Hospital of Medical School, Nanjing University, NO. 321, Zhongshan Road, Nanjing, China; 3 Department of Dermatology, Nanjing Jinling Hospital, Affiliated Hospital of Medical School, Nanjing University, Nanjing, China; 4 Department of Burns & Plastic Surgery, Nanjing Drum Tower Hospital Clinical College of Nanjing Medical University, NO. 321, Zhongshan Road, Nanjing, Jiangsu China

**Keywords:** Tension-reducing suture, LBD suture, Hypertrophic scar, Scar management

## Abstract

**Purpose:**

To investigate the application and effectiveness of tension-reducing suture in the repair of hypertrophic scars.

**Methods:**

A retrospective analysis of clinical data was conducted on 82 patients with hypertrophic scars treated at the Department of Burns and Plastic Surgery of Nanjing Drum Tower Hospital from September 2021 to December 2022. Patients were operated with combination of heart-shaped tension-reducing suturing technique and looped, broad, and deep buried (LBD) suturing technique or conventional suture method. Outcomes of surgical treatment were assessed before and 6 months after surgery using the Patient and Observer Scar Assessment Scale (POSAS) and the Vancouver Scar Scale (VSS).

**Results:**

Improvements were achieved on scar quality compared to that preoperatively, with a reduction in scar width (1.7 ± 0.6 cm vs. 0.7 ± 0.2 cm, *P* < 0.001). Assessment using the POSAS and VSS scales showed significant improvements in each single parameter and total score compared to preoperative values (*P* < 0.05). The Combination method group achieved better score in total score of VSS scale, in color, stiffness, thickness and overall opinion of PSAS scale, and in vascularity, thickness, pliability and overall opinion of OSAS scale.

**Conclusion:**

The amalgamation of the heart-shaped tension-reducing suturing technique and the LBD suturing technique has shown promising outcomes, garnering notably high levels of patient satisfaction in the context of hypertrophic scar repair. Patients have exhibited favorable postoperative recoveries, underscoring the clinical merit and the prospective broader applicability of this approach in the realm of hypertrophic scar management.

**Supplementary Information:**

The online version contains supplementary material available at 10.1186/s12893-024-02390-7.

## Introduction

Hypertrophic scars, also known as proliferative scars, represent a cutaneous pathology characterized by excessive proliferation and repair of new connective tissue in the dermis and deep tissues of the human body following injury [1]. Hypertrophic scars typically manifest as raised, smooth, and hyperpigmented masses that align with the original wound or incision site. Based on clinical characteristics, they can be categorized into linear hypertrophic scars or expansively growing hypertrophic scars [2]. In the early stages of hypertrophic scars, patients commonly experience pain and itching, while over time, these scars tend to darken in color and undergo volume reduction. Evidence suggests that individuals of Asian descent are more prone to the development of hypertrophic scars compared to Caucasians [3]. Unlike keloids, hypertrophic scars are confined to the original dimensions of the wound, characterized primarily by the deposition of type III collagen. They may gradually ameliorate over the course of several years. However, due to their specific locations in some patients, scar growth can not only impact aesthetics but also potentially induce muscle contracture, leading to joint functional impairments [4]. In clinical practice, there are primarily three approaches for the treatment of hypertrophic scars, namely pharmacological therapy, physical therapy, and surgical intervention. However, due to the incomplete elucidation of the pathogenesis of hypertrophic scars, it remains challenging to achieve complete scar resolution through a single treatment modality. Suboptimal treatment outcomes and recurrences continue to be a concern following therapy. In the realm of factors influencing the quality of incision healing and the subsequent formation of late scars, incision tension emerges as a pivotal determinant [5]. To mitigate this tension, we have implemented a fusion of the heart-shaped tension-reducing suture [6] in conjunction with the looped, broad, and deep buried (LBD) suturing technique [7, 8] to investigate the impact of tension-reduction sutures on the surgical treatment of hypertrophic scars. The results of the operation were analyzed retrospectively and compared with the conventional suture method.

## Patients and methods

### Patients

The patients were retrospectively enrolled from the Department of Burns and Plastic Surgery of Nanjing Drum Tower Hospital from September 2021 to December 2022 who were diagnosed as hypertrophic scar rather than and subjected to surgical treatment. Hypertrophic scars may have congestive edema, bright colors, and protrude the surface of the skin, which generally does not cause damage to the boundary skin. Keloids are usually dark purple, hard lumps that are significantly higher than the surface of the skin and may invade normal skin. Inclusion criteria: (I) Age: 18–65 years old. Exclusion: (I) Contraindications to surgery; (II) Subjected to laser or other non-operative treatment before; (III) Women during pregnancy, lactation and menstruation; (IV) Other systemic diseases; (V) Lost to follow up. This study was approved by the Ethics Committee of Nanjing Drum Tower Hospital (2020–10,901). The study was in accordance with the Declaration of Helsinki. Informed consents were obtained from all participants.

### Surgical methods

We designed the incision line along the scar perimeter using methylene blue as a marker. After achieving effective local anesthesia with 2% lidocaine (containing 1:200,000 epinephrine), we made an incision through the skin and subcutaneous tissue along the methylene blue-marked line, completely excising the scar. Subcutaneous tissue was trimmed to create an isosceles trapezoidal cross-section of the incision, with a narrower top and a wider bottom. The dermal layer was carefully approximated in advance to eliminate excess physical space, ensuring adequate hemostasis at the wound site.

#### Combination of the heart-shaped tension-reducing suturing technique and the LBD suturing technique

**Fig. 1:**
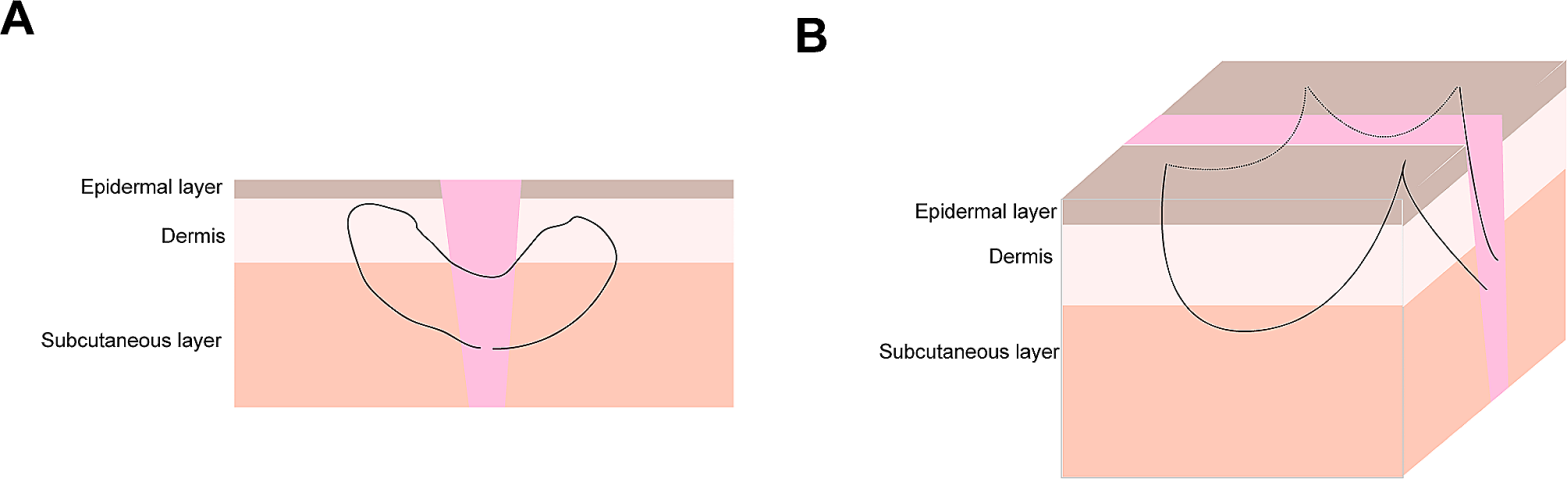
A schema describing this combined technique. (**A**) The heart-shaped tension-reducing suturing technique; (**B**) The LBD suturing technique

In accordance with the tension of the skin, 4 − 0 or 5 − 0 PDS sutures were selected for the implementation of a heart-shaped tension-reducing closure technique. During suturing, the needle tip was oriented upwards, penetrating through the fascial layer, following the needle’s trajectory. The suture traversed through the dermal layer in a bow-shaped ‘⌒’ pattern. Subsequently, it exited at the boundary between the subcutaneous fat layer and the subdermal layer. The procedure involved a mirrored operation for suturing the opposite skin edge, with knot tying performed below the fat layer (Fig. 1A).

For the LBD tension-reducing suturing technique, we first marked entry and exit points on both sides of the incision using methylene blue. These points were spaced approximately 1 cm apart on the same side and approximately 1 cm from the incision edge. Depending on the skin tension, 4 − 0 or 5 − 0 PDS sutures were chosen. The needle was inserted from within the incision, exiting at one of the marked points on the same side. Then, it re-entered at the same exit point and traversed through the entire thickness of the skin to the subdermal layer. Afterward, it exited at the second marked point approximately 1 cm away on the same side. The needle was re-inserted at this point, passing through the full skin thickness to the subdermal layer at a position 1 cm away on the same side. This process was repeated on the opposite side of the incision, mirroring the suturing pattern to form a rectangular trajectory. Finally, the sutures on both sides were conventionally knotted, ensuring complete and snug apposition of the skin edges with a mild everted tendency. The knots were buried deeply beneath the subcutaneous tissue after suturing (Fig. 1B).

#### Conventional suture method

In conventional suture method, an interrupted suturing method was commonly employed. The suture needle was introduced through the subcutaneous fascia, traverses the dermal layer to emerge, subsequently entered the contralateral dermal layer, exited through the fascial layer, and finally a knot was tied beneath the fascial layer.

Under tension-free conditions, interrupted sutures of 5 − 0 or 6 − 0 Prolene were used to close the surgical skin incision. Dressings were changed every 2 days postoperatively. Sutures were removed between 7 and 14 days post-surgery, depending on the surgical site. Following suture removal, the surgical area was regularly treated with silicone gel for 3–6 months.

### Outcomes evaluation

#### Incision complications

Postoperatively, monthly follow-up assessments were conducted to record whether patients experienced incision-related complications such as redness, swelling, infection, dehiscence, or fat liquefaction.

#### Scar hypertrophy assessment

Scar hypertrophy was assessed at 6 months post-surgery. Measurements of scar length and width were taken and compared to preoperative scar dimensions.

#### Scar quality evaluation

Preoperatively and at 6 months post-surgery, the Vancouver Scar Scale (VSS) and the Patient and Observer Scar Assessment Scale (POSAS) were employed to assess the incision recovery of all patients, thereby determining the effectiveness of scar repair.

The VSS is currently the most widely used scale for scar quality assessment. It comprises six variables: pigmentation, vascularity, pliability and height. Higher scores indicate more severe scar hypertrophy [9, 10].

The POSAS scale is the most comprehensive scar assessment tool currently in use. It consists of two parts: the patient scale and the observer scale. Both parts are scored using numeric values. Patients evaluate six parameters: pain, itch, color, stiffness, thickness, and irregularity of the scar compared to the surrounding normal skin. Observers assess five parameters, namely scar vascularity, pigmentation, pliability, thickness, and scar surface relief. Similarly, higher scores indicate more severe scar hypertrophy [11, 12].

### Statistical analysis

Statistical analysis was performed using IBM SPSS version 26.0 (IBM Corp., Armonk, NY). Normality distribution was assessed by evaluating frequency histograms and conducting the Shapiro-Wilk test. Paired sample t-test was applied to analyze pre and postoperative statistic of the same group, when comparing the conventional and combination groups the independent sample t test was employed. Pearson’s chi-squared test, correction tests, and Fisher’s exact probability test were employed to analyze categorical data. Quantitative data were presented as x ± s (mean ± standard deviation), while categorical data were expressed as percentages or rates. A two-tailed significance level was set at 0.05.

## Results

### Patient information

A total of 109 patients underwent tension-reducing suture surgery, of which 27 were lost to follow-up and thus excluded from the study. This resulted in the inclusion of 82 patients for analysis. Basic patient information including gender, age and scar location was shown in Table 1, and the two groups showed no differences.

**Table 1:** Baseline data of both groups

Characteristics	Total (*N* = 82)	Conventional method (*N* = 36)	Combination method (*N* = 46)	*P* value
Gender (male vs. female)	31:51	14:22	17:29	0.856
Age (mean ± SD)	34.1 ± 14.7	35.7 ± 15.2	31.6 ± 14.2	0.212
**Location site**				0.854
Head & neck	16	7	9	
Trunk	50	21	29	
Extremities	16	8	8	

Pearson’s chi-squared test was applied for parameter “Gender” and “Location site”. Independent sample t test was applied for parameter “Age”

### Outcome evaluation

**Fig. 2:**
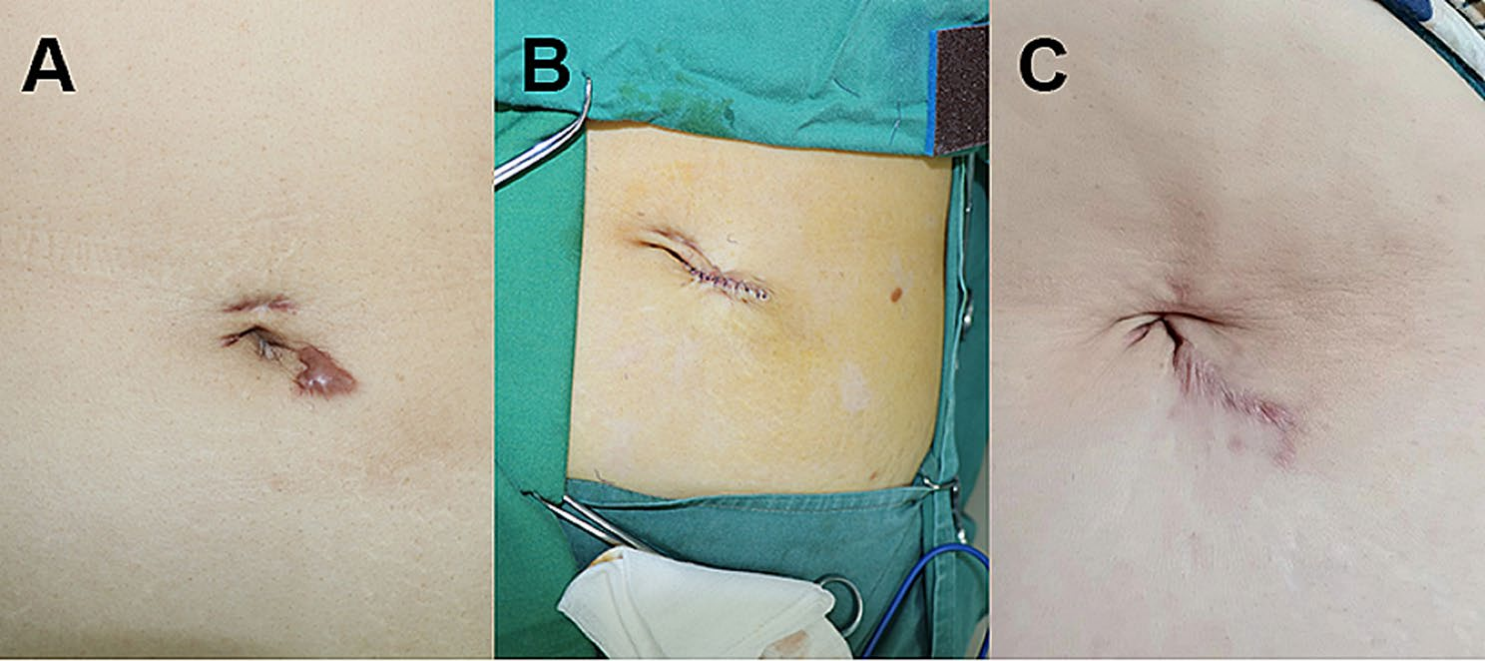
Comparison of an abdominal scar from a 56-year-old woman before and after surgical treatment with tension-reducing suture. (**A**) Before operation; (**B**) Immediately after surgery; (**C**) 6 months after operation

All 82 patients undergoing operation exhibited no instances of infection, dehiscence, or fat liquefaction. At the 6-month postoperative evaluation, none of the 82 patients showed scar hypertrophy (scar thickness > 2 mm). Comparing postoperative to preoperative scar characteristics, length increased, while width decreased, as shown in Table 2. Patients reported subjective symptom improvement, particularly in itch and pain. There was no significant difference in the length and width of preoperative scar between the two groups. The width of scar after operation in both groups was narrower than that before operation, but there was no difference in shortening the width of scar between the two groups. Figure 2 illustrates one typical case example of Combination method and Supplementary Fig. 1 shows the appearance of one case of Conventional method.

**Table 2:** Scar parameters before and after operation

	Conventional method			Combination method			
	Preoperation	Postoperation	Difference	Preoperation	Postoperation	Difference	*p* value
Width(cm)	1.9 ± 0.7	0.9 ± 0.3	1.0 ± 0.5	1.7 ± 0.6	0.7 ± 0.2	1.0 ± 0.4	> 0.99
Length(cm)	4.1 ± 3.7	7.4 ± 3.7	3.3 ± 3.7	4.4 ± 3.6	7.4 ± 3.7	3.0 ± 3.6	0.712

Independent sample t test was applied

At the 6-month postoperative follow-up of patients undergoing the heart-shaped tension-reducing suturing technique and the LBD suturing technique, according to the VSS scale, there were significant improvements compared to preoperative assessments in various aspects, including pigmentation (1.8 ± 0.7 vs. 1.0 ± 0.2, *P* < 0.001), vascularity (1.8 ± 0.9 vs. 0.7 ± 0.3, *P* < 0.001), pliability (1.9 ± 1.5 vs. 0.4 ± 0.3, *P* < 0.001), and height (1.3 ± 0.9 vs. 0.2 ± 0.2, *P* < 0.001). Overall scores were also significantly lower postoperatively compared to preoperative scores (8.5 ± 4.1 vs. 3.6 ± 1.0, *P* < 0.001). In the Conventional method group, there were also significant improvements compared to preoperative assessments in various aspects, including pigmentation (1.7 ± 0.7 vs. 1.0 ± 0.3, *P* < 0.001), vascularity (1.9 ± 1.0 vs. 0.9 ± 0.4, *P* < 0.001), pliability (1.7 ± 1.6 vs. 0.5 ± 0.5, *P* < 0.001), and height (1.5 ± 1.0 vs. 0.4 ± 0.5, *P* < 0.001). Overall scores were also significantly lower postoperatively compared to preoperative scores (6.8 ± 3.5 vs. 2.8 ± 1.0, *P* < 0.001). Although there was no difference in each specific parameter, the Combination method group had more advantages in reducing the total score of scar (Table 3).

**Table 3:** VSS score before and after operation

	Conventional method			Combination method			
	Preoperation	Postoperation	Difference	Preoperation	Postoperation	Difference	*p* value
Pigmentation	1.7 ± 0.7	1.0 ± 0.3	0.7 ± 0.5	1.8 ± 0.7	1.0 ± 0.2	0.8 ± 0.5	0.371
Vascularity	1.9 ± 1.0	0.9 ± 0.4	1.0 ± 0.7	1.8 ± 0.9	0.7 ± 0.3	1.1 ± 0.6	0.488
Pliability	1.7 ± 1.6	0.5 ± 0.5	1.2 ± 1.0	1.9 ± 1.5	0.4 ± 0.3	1.5 ± 1.0	0.181
Height	1.5 ± 1.0	0.4 ± 0.5	1.1 ± 0.7	1.3 ± 0.9	0.2 ± 0.2	1.1 ± 0.5	> 0.99
Total	6.8 ± 3.5	2.8 ± 1.0	4.0 ± 1.2	6.6 ± 3.1	2.0 ± 0.7	4.6 ± 1.4	0.044^∗^

VSS, Vancouver scar scale. Independent sample t test was applied

^*^P< 0.05

According to the POSAS scale, the PSAS component results of patients subject to tension-reducing suture indicated significant improvements postoperatively compared to preoperative assessments in color (5.4 ± 2.6 vs. 3.0 ± 1.4, *P* < 0.001), stiffness (4.7 ± 2.6 vs. 1.4 ± 0.4, *P* < 0.001), thickness (4.6 ± 2.8 vs. 1.5 ± 0.5, *P* < 0.001), regularity (3.1 ± 2.2 vs. 2.3 ± 0.8, *P* = 0.025), pain (3.7 ± 2.0 vs. 1.7 ± 0.6, *P* < 0.001) and itch (3.9 ± 2.2 vs. 1.7 ± 0.7, *P* < 0.001). Overall assessment was also better than preoperative evaluations (5.8 ± 2.0 vs. 2.1 ± 0.5, *P* < 0.001). In the OSAS component, there were significant improvements postoperatively compared to preoperative assessments in vascularity (7.4 ± 2.1 vs. 2.9 ± 1.3, *P* < 0.001), thickness (4.9 ± 2.2 vs. 1.3 ± 0.3, *P* < 0.001), pigmentation (5.2 ± 2.9 vs. 3.9 ± 1.8, *P* = 0.015), pliability (5.1 ± 2.7 vs.1.5 ± 1.0, *P* < 0.001), and relief (2.6 ± 1.8 vs. 1.0 ± 0.6, *P* < 0.001). Overall assessment (6.2 ± 2.1 vs. 1.7 ± 0.9, *P* < 0.001) also showed significant improvement. The Conventional method group also showed significant improvements postoperatively in each parameter of PSAS and OSAS. However, in PSAS scale, the combination group showed better results in scar parameter reduction including color, stiffness, thickness and overall opinion. In OSAS scale, the combination group also got better parameter reduction in vascularity, thickness, pliability and overall opinion (Table 4).

**Table 4:** POSAS score before and after operation

	Conventional method			Combination method			
	Preoperation	Postoperation	Difference	Preoperation	Postoperation	Difference	*p* value
**PSAS**							
Color	5.1 ± 3.1	3.7 ± 1.7	1.4 ± 1.3	5.4 ± 2.6	3.0 ± 1.4	2.4 ± 1.8	0.006^∗^
Stiffness	4.9 ± 2.3	1.9 ± 0.6	3.0 ± 1.3	4.7 ± 2.6	1.4 ± 0.4	3.3 ± 1.3	0.303
Thickness	5.0 ± 2.9	1.9 ± 0.9	3.1 ± 1.8	4.6 ± 2.8	1.5 ± 0.5	3.1 ± 1.7	> 0.99
Irregularity	2.9 ± 2.3	2.6 ± 1.1	0.3 ± 0.2	3.1 ± 2.2	2.3 ± 0.8	0.8 ± 0.6	< 0.001^∗^
Pain	3.8 ± 2.3	1.6 ± 0.5	2.2 ± 1.2	3.6 ± 2.0	1.7 ± 0.6	1.9 ± 1.1	0.242
Itch	3.7 ± 2.0	1.5 ± 0.5	2.2 ± 1.1	3.9 ± 2.2	1.7 ± 0.7	2.2 ± 1.2	> 0.99
Overall opinion	5.5 ± 2.3	2.8 ± 0.9	2.7 ± 0.9	5.8 ± 2.0	2.1 ± 0.5	3.7 ± 1.1	< 0.001^∗^
**OSAS**							
Vascularity	7.1 ± 2.0	3.9 ± 1.7	3.2 ± 1.3	7.4 ± 2.1	2.9 ± 1.3	4.2 ± 1.5	0.002^∗^
Thickness	5.1 ± 2.4	1.5 ± 0.5	3.6 ± 1.3	4.9 ± 2.2	1.3 ± 0.3	3.6 ± 1.2	> 0.99
Pigmentation	4.7 ± 2.7	4.2 ± 1.9	0.5 ± 0.3	5.2 ± 2.9	3.9 ± 1.8	1.3 ± 0.8	< 0.001^∗^
Pliability	5.4 ± 2.9	2.2 ± 1.8	2.8 ± 1.2	5.1 ± 2.7	1.5 ± 1.0	3.6 ± 1.5	0.011^∗^
Relief	2.3 ± 2.0	1.1 ± 1.2	1.2 ± 1.1	2.6 ± 1.8	1.0 ± 0.6	1.6 ± 1.1	0.106
Overall opinion	5.8 ± 2.4	2.2 ± 1.3	3.6 ± 1.9	6.2 ± 2.1	1.7 ± 0.9	4.5 ± 1.9	0.036^∗^

POSAS, patient and observer scar assessment scale; PASA, patient scar assessment scale; OSAS, observer scar assessment scale. Independent sample t test was applied

^*^P < 0.05

## Discussion

The formation of hypertrophic scars is a complex process influenced by various factors, making it challenging to achieve improvement through short-term pharmaceutical and physical interventions. An increasing number of patients are opting for surgical excision to address this issue. However, postoperative recurrence of scar hypertrophy, can significantly impact patient satisfaction. Research has shown that postoperative incisional tension plays a pivotal role in both healing and scar formation. The prolonged effect of tension at the surgical site stimulates surrounding tissues, exacerbates inflammatory responses, promotes the formation of new blood vessels within granulation tissue, leading to the local synthesis of excessive collagen [13]. These processes are unfavorable for scar healing and can persist for several months, significantly impairing the postoperative scar’s recovery. Consequently, tension-reducing sutures postoperatively have shown favorable outcomes in enhancing surgical efficacy and improving postoperative scar formation [14–16].

While traditional suturing techniques may offer shorter closure times, the failure to adequately release tension around the incision site often leads to the formation of deep dead spaces postoperatively. This can result in complications such as infection, seroma formation, wound dehiscence, and delayed healing, all of which can contribute to scar recurrence. Consequently, conventional suturing methods are no longer sufficient to meet the demands of scar repair [17]. With increasing patient expectations for skin aesthetics, recent years have witnessed a continuous evolution of tension-reducing suturing techniques including primary layered closure, the buried vertical mattress suture (BVMS), modified BVMS, set-back buried dermal suture, and the butterfly suturing technique [14–18].

In this study, we employed the combination of heart-shaped tension-reducing sutures and LBD suturing technique. The heart-shaped tension-reducing suture is characterized by the trapezoidal wound trimming, which, when the sutures are tightened, results in a slight eversion of wound edges, allowing for a full approximation of the wound. This technique effectively eliminates deep dead spaces, reducing postoperative complications such as infection, seroma formation, and promoting faster healing. In recent years, it has found wide application in areas with high tension, particularly in obstetric and gynecologic procedures like cesarean sections. Studies by Ni [6] and colleagues have shown that the use of subcutaneous heart-shaped tension-reducing sutures in abdominal incisions after cesarean sections produces precise and cosmetically favorable closure results, significantly reducing scar formation and enhancing aesthetics. This technique is worthy of clinical promotion and application. To achieve thorough tension reduction, it is crucial to pay equal attention to the handling of both the dermal layer and the superficial fascial layer.

In recent years, Tang [8] have made multiple refinements to the LBD suturing technique, effectively balancing tension reduction with the increasing aesthetic demands of patients. When performing LBD sutures, the sutures partially traverse the dermal layer, allowing them to carry more dermal tissue. This facilitates the relaxation of wound edges by stretching a greater amount of dermal tissue, which, in turn, stabilizes tension around the wound, reduces peri-incisional dead spaces, and minimizes the occurrence of complications such as seroma and infection. Furthermore, this approach avoids skin damage and secondary scar formation associated with externalized sutures, leading to improved wound aesthetics, and aiding in achieving the desired scar repair outcomes. Additionally, with advancements in suture materials, new absorbable sutures like PDS possess enhanced tensile strength and flexibility while maintaining a longer presence in the body. They provide sustained tension reduction effects for 1–3 months until absorbed by the surrounding skin tissues.

In the surgical procedure, we made adjustments to the spacing of LBD sutures and increased the number of sutures in areas with higher tension, such as the limbs, to enhance tension reduction. However, it’s crucial to note that the distance from the needle to the wound should not be reduced, as this could elevate the risk of skin edge necrosis. Additionally, during the suturing process on both sides of the incision, a curved path within the dermal layer was considered, which helps distribute tension and effectively improves the appearance of the entry points in the skin, leading to higher patient acceptance of the early incision appearance. The statistical results of this study demonstrate the stable and reliable tension reduction effects of the LBD technique. It offers a more sustained and consistent tension reduction effect, particularly beneficial for wounds with higher tension and scar repair improvement.

In the treatment of keloid, postoperative radiotherapy within 48 h as a method to prevent recurrence has been widely recognized [19–21]. Extra- or intralesional excision of hypertrophic scars followed by early postoperative radiotherapy should be both simple and effective at preventing recurrence at excision sites. However, we need long-term results including carcinogenesis to apply it as a reliable medical intervention [22]. Considering this terrible side effect, our patients are not very receptive to radiotherapy, especially in people with hypertrophic scar rather than keloid. Therefore, the patients in this study did not receive adjuvant radiotherapy. However, a sufficiently safe method of radiotherapy is still worth advocating.

Although the combined method we describe can effectively reduce the tension of the wound. However, it also has some shortcomings, such as the extension of operation time, certain experience requirements for operators, and more sutures buried under the skin may increase the incidence of foreign body response and so on. The study has some limitations. First, this is a retrospective study, not a completely blind randomized controlled study. There may be bias when patients are assigned to the treatment group. Second, the scar scale we use are relatively objective parameters. Although they have been widely used, there may still be human deviation in the process of implementation. A completely objective method for evaluating scars has yet to be developed.

In summary, this study suggests that the combination of heart-shaped tension-reducing sutures and LBD tension-reducing sutures is an effective approach for repairing hypertrophic scars. It provides excellent tension reduction, resulting in higher postoperative aesthetic scores from patients. This technique is worthy of clinical promotion and application.

### Electronic supplementary material

Below is the link to the electronic supplementary material.

Supplementary Material 1

Supplementary Material 2

## Data Availability

No datasets were generated or analysed during the current study.
