# Supplementary figures and images for: Application and effect of tension-reducing suture in surgical treatment of hypertrophic scar
Source: BMC Surg. 2024 Apr 23;24:119. doi: 10.1186/s12893-024-02390-7 (PMC11036683; doi:10.1186/s12893-024-02390-7)

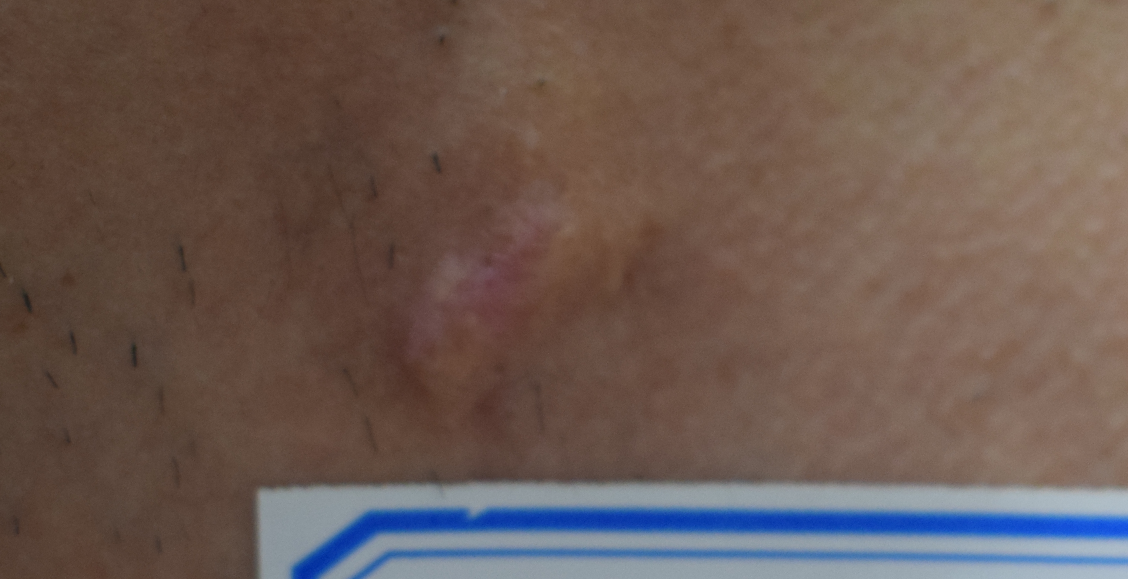

Supplement: Supplementary file 1 — Supplementary Material 1 [file 12893_2024_2390_MOESM1_ESM.tif]
