# Supplementary material for: Application and effect of tension-reducing suture in surgical treatment of hypertrophic scar
Source: BMC Surg. 2024 Apr 23;24:119. doi: 10.1186/s12893-024-02390-7 (PMC11036683; doi:10.1186/s12893-024-02390-7)
Supplement: Supplementary file 2 — Supplementary Material 2 [file 12893_2024_2390_MOESM2_ESM.docx]

**Supplementary Figure 1**

A picture of mandibular scar from a 34-year-old man undergoing conventional method.
